# How Can We Actually Change Help-Seeking Behaviour for Mental Health Problems among the General Public? Development of the ‘PLACES’ Model

**DOI:** 10.3390/ijerph19052831

**Published:** 2022-02-28

**Authors:** June S. L. Brown, Stephen Lisk, Ben Carter, Sharon A. M. Stevelink, Ryan Van Lieshout, Daniel Michelson

**Affiliations:** 1 Psychology Department, Institute of Psychiatry, Psychology and Neuroscience (IoPPN), Denmark Hill Campus, Kings College London, London SE5 8AF, UK; stephen.lisk@kcl.ac.uk; 2 Biostatistics Department, Institute of Psychiatry, Psychology and Neuroscience (IoPPN), Denmark Hill Campus, Kings College London, London SE5 8AF, UK; ben.carter@kcl.ac.uk; 3 Department of Psychological Medicine, Institute of Psychiatry, Psychology and Neuroscience (IoPPN), Denmark Hill Campus, Kings College London, London SE5 8AF, UK; sharon.stevelink@kcl.ac.uk; 4 Department of Psychiatry and Behavioural Neurosciences, McMaster University, Hamilton, ON L8S 4L8, Canada; vanlierj@mcmaster.ca; 5 Psychology Department, Sussex University, Brighton BN1 9RH, UK; daniel.michelson@sussex.ac.uk

**Keywords:** facilitators, depression, stress, adults, adolescents, stigma, psychoeducational workshops, early intervention, treatment gap

## Abstract

Good treatment uptake is essential for clinically effective interventions to be fully utilised. Numerous studies have examined barriers to help-seeking for mental health treatment and to a lesser extent, facilitators. However, much of the current research focuses on changing help-seeking attitudes, which often do not lead to changes in behaviour. There is a clear gap in the literature for interventions that successfully change help-seeking behaviour among the general public. This gap is particularly relevant for early intervention. Here we describe the development of a new model which combines facilitators to treatment and an engaging, acceptable intervention for the general public. It is called the ‘**PLACES**’ (**P**ublicity, **L**ay, **A**cceptable, **C**onvenient, **E**ffective, **S**elf-referral) model of treatment engagement. It is based on theoretical work, as well as empirical research on a low intensity psychoeducational cognitive behavioural therapy (CBT) intervention: one-day workshops for stress and depression. In this paper, we describe the development of the model and the results of its use among four different clinical groups (adults experiencing stress, adults experiencing depression, adolescents (age 16–18) experiencing stress, and mothers with postnatal depression). We recorded high rates of uptake by people who have previously not sought help and by racial and ethnic minority groups across all four of these clinical groups. The clinical and research implications and applications of this model are discussed.

## 1. Introduction

It is estimated that 17% of the adult population in England have mental health problems [1]. However, only 30% of those affected individuals seek professional help [1]. Problems with low rates of help-seeking have been widely described [2] and can result in poorer mental health outcomes, including increased chronicity. Low help-seeking also leads to poorer recruitment and selection bias in research studies [3], raising the possibility that these findings may lack external validity. Given the importance of clinical treatment access for individuals, their families, and healthcare systems, it is important to understand the barriers to access. However, it is potentially even more important to identify and understand factors that can actually increase treatment uptake in the community.

Typically, research focuses on barriers and facilitators regarding attitudes around help-seeking. However, studies have had mixed success regarding interventions to improve help-seeking behaviour. A systematic review that was conducted assessed the success of randomised controlled trials (RCT) of interventions aimed at increasing help-seeking behaviour in adults with problems of depression, anxiety, and general distress [4]. Mental health literacy was the only intervention found to be associated with improved help-seeking attitudes, but this had no effect on help-seeking behaviour. The authors concluded, “significantly... very little is known about what interventions increase help-seeking behaviour”.

Another systematic review [5] examined the effectiveness of help-seeking interventions in changing attitudes, intentions, and behaviour. Differences in help-seeking behaviour were found, but only among those with mental illness and those at risk of mental illness. However, no changes in help-seeking behaviour were found among the general public or among children or adolescents. This is important because they will often experience some troubling symptoms but may be unsure about what these mean, may try to cope with the problems themselves or may feel reluctant to seek help from a professional.

This demonstrates a major gap in the literature regarding interventions aiming to improve help-seeking behaviour among the general public, specifically adults and children or adolescents. It is known that the onset of mental health problems for 75% of people occurs by age 24 [6]. To prevent these problems from arising or becoming chronic, it is vital we offer early intervention when symptoms are first experienced [7], whilst at school, college, or among people who may be unsure of their mental health status.

Therefore, in this paper, we present a model we have been using which describes how members of the public with problems can more easily access professional services. In developing this model, we were informed by the conceptual model of help-seeking by Gask [8], which uses a patient-centred approach. The “Community engagement” aspect of this model is particularly important as it encompasses the uncertainty of individuals first engaging in seeking help with processes such as ‘candidacy’ (should I be seeking help?), ‘navigation’ (where do I seek help from?) and ‘appearance’ (actually going to seek help). The delivery of tailored psychosocial interventions is equally important as these need to fit with the needs of a population (e.g., ethnic minorities) and the social and cultural aspects of the group.

Help-seeking is largely researched in relation to barriers rather than facilitators, with stigma being the most commonly researched barrier to help-seeking. Despite its attention, it is actually listed as only the 4th most common barrier [9]. However, the authors note that stigma is also highly likely to influence other barriers.

How individuals view their mental health problems is key, and low perceived need is another very common barrier [10,11,12]. Recent work conducted in a military population found that the perceived need for treatment (and not stigma) was the most prominent barrier to care [13]. Self-reliance is often found to be the preferred way of coping, particularly among young people [14].

Poor mental health literacy [15] invariably affects help-seeking; this refers to a limited understanding of mental health problems as well as not knowing where to go for help. A related problem is how the general public first seeks help: going to their general practitioner (GP) has been the standard way of accessing help in many countries but may act as a barrier for some racial and ethnic groups [16].

Finally, individuals may view treatment as not being very acceptable and may not take it up and/or drop out as a result [17]. For example, online interventions (especially if self-guided) have this problem [18]. Services can also be inconvenient when weekly sessions are offered during office hours between 9–5 pm and/or if delivered in formalised mental health settings [19].

The **‘PLACES’** model was initially developed in the context of developing large-scale stress workshops for a city-wide mental health promotion campaign [20] and then extended with depression workshops [21]. It was further tested with adolescents experiencing stress [22] and mothers with postnatal depression [23]. The interventions have also been shown to be effective and are separately reported for stress [24], depression [25], adolescents [26], and with mothers affected by postnatal depression [27].

## 2. The Development and Rationale for Facilitating Factors in ‘PLACES’ Model

This is a summary paper of the treatment engaging factors that we have developed and utilised. We aim to synthesise our studies covering the different clinical areas, as well as different sociodemographic groups, into one paper. This paper will report on how successful the model has been at (a) engaging total numbers of participants, (b) attracting non-consulters who had not consulted their general practitioners (GPs) or professional services, (c) those with severe problems, and (d) engaging those from racial and ethnic minority groups.

### 2.1. Stress Workshops

Context

The large-scale workshops were part of a city-wide mental health promotion campaign. The aims of the workshops were:

(1) To be as accessible as possible, especially to non-consulters.
(2) To offer an acceptable large-scale intervention.

The stress workshop intervention was a ‘low-intensity’, large-scale, psychoeducational day-long cognitive behavioural therapy (CBT) workshop for up to 30 people. It was designed to be brief and as clinically accessible as possible, with few barriers to help-seeking. The content of the programme was informed by the evidence-based principles of CBT [24]. Because of the possible stigmatising effects of diagnostic labels, the wording of the programme (and its publicity) avoided the use of such words as ‘anxiety’ and ‘depression’. Instead, words such as ‘stress’ were used.

**Figure 1:**
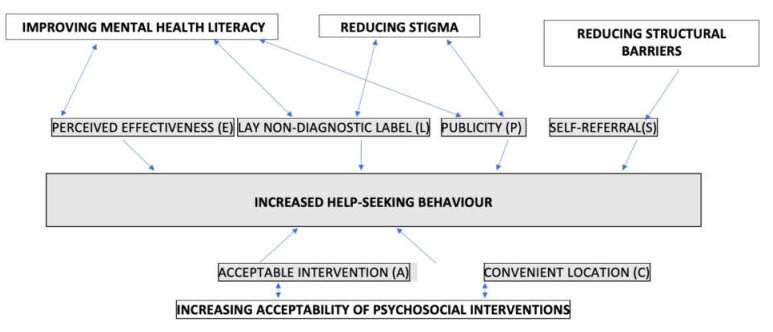
Relationship of “PLACES’ model and common barriers to help-seeking.

Based on the literature, it was decided to use four methods to engage the public: self-referral (S), publicity (P), acceptable (and engaging) intervention (A), and convenient location (C). Figure 1 describe how these methods relate to common barriers accessing services, namely mental health literacy [15], stigma [9], and structural barriers [16], as well as intervention barriers such as the acceptability [17] and convenience [28] of the intervention.

*1.*  *SELF-REFERRAL (S)*

GP referrals have been the standard way of accessing help in many countries. However, there are significant problems with access regarding some racial and ethnic minority groups (e.g., South Asian, black Caribbean, African) who are often reluctant to consult their GP [16].

A self-referral route was therefore developed for the workshops to allow easier access [29]. This involved publicising the workshops widely in different community settings, with contact details highlighted to enable direct access.

*2.*  *PUBLICITY (P)*

Members of the public often struggle with aspects of mental health literacy, including not knowing what mental health problems are or if treatments will be effective, both of which are aspects of mental health literacy [15]. Furthermore, self-reliance, or trying not to ask for help from others, is a common barrier [10].

The aim of using detailed publicity was, therefore, to draw attention to the workshops and utilise a more attractive and engaging method to inform the public that more positive coping strategies were possible. Colourful and striking flyers were used to widely publicise workshops in libraries, pharmacies, community centres, and GP surgeries. Feedback about this approach was very positive [30].

*3.*  *ACCEPTABLE (AND ENGAGING) INTERVENTION (A)*

Acceptability of the treatment/intervention is another key barrier [17]. Computerised CBT has not been found to be acceptable by many participants [18], especially when self-guided. The take-up rate is sometimes as low as 50%, with high rates of dropout. Indeed, a scoping review [30] found that e-mental health treatment services were perceived as less helpful than traditional face-to-face interventions.

The stress workshops were designed to be run over one day and delivered in a group format to allow participants to share experiences without this process distracting from the psychoeducational focus. It was also designed around the concentration span of 20 min [31], with varied activities, including small and large group discussions, demonstrations of methods in role-plays by leaders, and individual exercises [24]. The workshop was not specifically adapted to the needs of minority groups but was responsive to the different groups who attended. A colourful workbook covering the day’s programme was provided to help participants sustain their progress.

*4.*  *CONVENIENCE—SETTING AND TIMING (C)*

Services can be **inconvenient** when run during office hours between 9–5 pm and/or in formalized mental health settings [19]. Weekly sessions during office hours may not suit everyone; participants may live far away from the therapy setting or may feel under pressure to get better quickly. Some patients also find it difficult to engage in lengthy psychological treatment and may prefer more intensive shorter treatments [32]. There is surprisingly little literature around this topic.

Stress workshops were, therefore, run in a community setting, using leisure centres at the weekend to improve convenience and reduce possible stigma. The centre was conveniently situated for buses and car parking. While different, the move to more online interventions because of the pandemic is also important because it demonstrates the place of convenience [33].

Empirical outcomes of the stress workshops:

***(1)***  ***Uptake:*** 176 attended the information meeting.
***(2)***  ***Non-consultation:*** Just under half (41%) had not previously consulted their GPs [20].
***(3)***  ***Severity***: In this study, participants’ anxiety scores were above average (Spielberger trait scores 51.5), which is higher than the threshold for probable anxiety [34]. A finding from a later study of Stress participants indicated 66% of stress workshop participants scored above the ICD psychiatric threshold [35].
***(4)***  ***Ethnicity:*** Data was not collected in the initial study but was collected in a later study in London, where 13.2% reported themselves as Black (9.4%) or Asian (3.8%).

### 2.2. Depression Workshops

Context

When we used this initial model with pilot depression workshops, virtually all of the participants who attended had already been diagnosed as having depression and were being treated in primary or secondary care [36]. We, therefore, added two new factors: using more lay non-diagnostic titles (**L**) and putting emphasis on the perceived effectiveness of the programme (**E**). These two new factors were added to Figure 1. We also changed the acceptability (**A**) of the programme by altering the description from being a depression programme to one on self-esteem/confidence.

*1.*  *LAY NON-DIAGNOSTIC TITLES (L)*

Mental health literacy can have positive and negative effects. While it can be very helpful in providing an understanding of mental health problems, diagnostic labels can also act as a barrier to treatment and deter some people [37]. Furthermore, a review of the effects of stigma and school interventions showed that the labelling of interventions compromised efforts to increase access to targeted school-based interventions [38]. A number of studies show that an individual’s **perception of the problem** is the most common barrier [10], with problems often perceived as ‘social’ (e.g., problems of living) rather than as ‘medical’ or ‘psychological’ [39].

As ‘depression’ could be seen as a stigmatising diagnostic term, we, therefore, changed the title of the intervention from ‘depression’ to ‘self-confidence’. This decision was also based on the close relationship between depression and self-esteem [40]. The term ‘self-confidence’ was used rather than ‘self-esteem’ because the former is more often used colloquially and is more understandable to the public. The language used for publicising interventions could have a positive effect on help-seeking. We have described this approach as “sensitively engaging” in a previous paper [41].

*2.*  *PERCEIVED) EFFECTIVENESS OF INTERVENTION (E)*

When stigma was carefully examined [9], ‘treatment stigma’ (stigma specifically associated with seeking or receiving treatment for mental ill-health) was found to be one of the strongest predictors of low help-seeking. Among young people, the belief that the treatment would be effective was also shown to affect their willingness to seek help [14]. Furthermore, this group perceived possible benefits as being more important than stigma-related factors [42]. Perceived effectiveness of treatment was also found to influence seeking formal help among men [43].

In the publicity, it was therefore decided to market the potential effectiveness of the workshops more directly—“Do you want to believe in yourself more? Handle times when things don’t go your way? Be more effective in what you do? Put yourself down less often?” [3].

*3.*  *ACCEPTABILITY OF PROGRAMME (A)*

We also made changes to the programme to make it more acceptable (A). The content of the programme was changed to a CBT programme of self-confidence, described in Horrell [25], based on Fennell [44]. The engaging format was again used with 20-min periods for the different methods, with interaction where possible. A colourful workbook was again provided to remind participants of the programme.

**Other facilitators** were largely kept the same; publicity (P), self-referral (S), and convenience (C).

Empirical outcomes of depression workshops:

***Uptake*** Changing the title from ‘depression workshops’ to ‘self-confidence workshops’ led to a marked increase in recruitment from 28 to 120 attendees [21].

***Non-consultation*** After the change, the proportion of people who had not previously sought help increased from 9.8% [37] to 39% [21]. This suggests that depression is a term used by people already accessing mental health services.

***Severity of problems*** In total, 39% of those who attended the initial self-confidence workshops had not previously consulted their GPs about depression [21]. An important finding from a later diagnostic study of self-confidence participants was that 72.6% (n = 106) of the workshop scored above the ICD psychiatric threshold [35].

***Equity*** Self-confidence workshops did successfully engage ethnic minority groups, and the proportion was representative of the local population [21]; in particular, 35.2% reported themselves to be black (28.3%) which matches the ethnicity of the surrounding area, which was 25.9% black. This indicates more equitable access.

Testing of ‘PLACES’ model

Given the success of the stress and self-confidence workshops in engaging participants, we decided to use and test this model with other age and clinical groups.

### 2.3. Stress Workshops for Adolescents

Context

It was decided to adapt the adult version of the stress workshops for adolescents to offer early intervention and to test if this model could successfully engage adolescents who are traditionally reluctant to engage in services [45].

Extensive focus groups and interviews were conducted when adapting the workshops for adolescents [22]. There were two major changes with this group: convenience (C) and acceptability (A). Lay (L) titles were also utilised.

*1.*  *CONVENIENCE—SETTING AND TIMING (C)*

In the pilot study, attendance of the workshops was greater when these were run in schools compared to community settings such as libraries, youth clubs, or community centres. Schools were seen as safer, more convenient, and familiar environments to the adolescents than the other settings [22,46].

*2.*  *ACCEPTABLE (AND ENGAGING) INTERVENTION (A)*

Strong preferences were expressed for more interactive and engaging content. It was also felt that a more individualised approach, focussing on goal planning, would be helpful in addition to the day-long workshop. A pre-workshop planning session, plus up to three phone calls after the workshop, were added.

*3.*  *LAY NON-DIAGNOSTIC TITLES (L)*

Adolescents suggested that the programme should be called the ‘DISCOVER’ workshop rather than just stress workshops [22].

Some other facilitators were adapted, as outlined below.

*4.*  *SELF-REFERRAL (S)*

As self-reliance has been shown to be particularly relevant to adolescents [14], self-referral was seen as particularly valuable and emphasised.

*5.*  *PUBLICITY (P)*

As well as paper publicity, popular social media platforms were also used [26].

Empirical outcomes of adolescent workshops:

***Uptake:*** Thirty-three participated in the pilot workshops [22] and 155 attended the feasibility trial [26].

***Non-consultation*** About 70% were non-consulters, 73.3% [22] and 69.7% respectively [26];

***Equity.*** The rate for racial and ethnic students was high: 64.5% [22] and 57.4% [26].

***Severity*** Just under 50% scored above the threshold for anxiety problems on the Revised Child and Anxiety Scale (RCADS)-anxiety subscale, and just over 25% scored over the threshold for depression on the MFQ [26]. In the pilot study, 74.2% scored above one or both of these clinical cut-offs [22].

### 2.4. Postnatal Depression (PND) Workshops

Context

Day-long PND workshops were developed as a result of recognising that, due to the small number of psychological therapists available, only a small proportion of mothers with PND (15%) were able to receive any evidence-based care [47]. Long waitlists for psychotherapy, women’s preferences for it over medication, a lack of time, and a reluctance to travel to regular appointments have been shown as substantial barriers [48].To increase engagement, all aspects of the **‘PLACES’** model were used but with some variations.

*1.*  *PERCEIVED EFFECTIVENESS (E)*

CBT techniques were used and capitalised on the fact that postpartum women prefer psychotherapies over medication [49]. This highlighted that the workshop utilising non-pharmacological techniques was seen as important in increasing uptake, particularly among lactating mothers.

*2.*  PUBLICITY AND SELF-REFERRAL (P and S)

Even though speciality psychiatric care in Canada normally requires a referral from a GP or another physician [23], the majority of the women self-referred (90–95%) to the workshop (and 80% were recruited via social media alone) [23].

*3.*  *CONVENIENCE (C)*

The pilot face-to-face postnatal workshops were held in convenient locations on transit routes and in non-medical settings (community centres, libraries, etc.). Following consultation, these were run during the week as women with young children said they preferred weekday workshops, allowing more time with partners in the evenings or at weekends.

*4.*  *ACCEPTABILITY (A)*

Extensive clinical work with mothers, collaboration with relevant public health, and community organizations helped to define workshop content, structure, and materials [23,27].

Empirical outcomes of PND workshops

***Uptake:*** Eighteen postnatally depressed women participated in the first pilot study [23]. The online treatment involved 403 mothers [27].

***Non-consultation:*** Over half had not previously sought help; pilot 57% [23] and online 55%, respectively [27].

***Severity***: All women had PND in both the pilot and online studies, scoring over 10 on Edinburgh Postnatal Scale (EPDS) [23,27].

## 3. Discussion

We have presented the implementation findings of the **‘PLACES’** model (**P**ublicity, **L**ay, **A**cceptable, **C**onvenient (Perceived) **E**ffectiveness, **S**elf-referral) of treatment engagement. The model incorporates both referral facilitators as well as intervention factors for improving engagement with mental health services/support. Four of the factors focus on the referral and publicising of the interventions (**P**ublicity, **L**ay titles, (Perceived) **E**ffectiveness, and **S**elf-referral), and the other two factors relate to the intervention and its delivery (**A**cceptable and **C**onvenient).

This success of the model (with adaptations from the original stress workshop format) is that it has been shown to be effective in engaging people affected by depression, adolescents, those who have been traditionally difficult to engage, as well as mothers affected by PND who sometimes struggle to access to evidence-based psychological help.

The **‘PLACES’** model is also very relevant to early intervention [7]. It helps members of the public (whether adults or adolescents) think about their mental health needs, particularly when they are unsure about what to do next. This is particularly important because ‘perceived need’ has been found to be a key barrier to help-seeking. Given mental health services tend not to be publicised much, we believe that key elements have been the relevant ‘social marketing’ aspects: publicity (P) highlighting mental health problems in a more ‘normal’ way to lay people (L), highlighting the perceived effectiveness of the intervention (E), and setting up a self-referral system (S).

Secondly, it tries to offer ‘tailored’ and effective psychosocial interventions that are acceptable (A) and convenient (C). Tailored interventions would ‘make more sense’ to those who would otherwise not use mental health services. Even though not widely researched, the acceptability of the intervention significantly affects take-up and dropout rates [17]. A recent review shows the relevance of adapted interventions for racial and ethnic minorities [50].

Convenience is another important facilitator within the PLACES model. Whereas this facilitator has been informed by and tested during pre-COVID-19 case studies, the huge drive towards the online delivery of mental health treatment to ensure continuity of care, and its possible effect on the implementation of our model, needs to be considered [33]. For some members of the public, the provision of online therapy has been a convenient change, whereas other people may have been excluded due to limited technological literacy, lack of resources, or preference for face-to-face sessions.

With self-referral, the **‘PLACES’** model has successfully engaged people from diverse backgrounds as well as those with serious problems [25] and has led to the Improving Access to Psychological Treatments (IAPT) service in the UK to adopt this method [51]. Furthermore, self-referral was found to attract a more equitable group from ethnic minorities and employment status compared to GP referrals [52]. A similar picture was found with a self-referral system in the military which was also found to have high rates of non-consulters (69%) as well as those with serious problems (72%) [53].

The proposed model is unique for several reasons. Firstly, it fills an important gap in the literature about the help-seeking behaviour of the general public. Two major reviews have concluded that effective methods that change the help-seeking behaviour of the general public have not been identified. The **‘PLACES’** model focuses on changing help-seeking ***behaviour*** rather than shifting attitudes alone [4]. Secondly, it uniquely combines facilitators with an engaging intervention for the general public. It describes more of a ‘bridge’ that a person with a mental health problem would cross in order to reach services that up to now have been avoided or are unknown. This model is, therefore, about trying to make the services more ‘reachable’. Thirdly, it is based on the wider literature, as well as empirical work conducted in developing the intervention of large-scale one-day psychoeducational workshops in the community. Finally, it aims to be clinically relevant so that services can apply these ‘bridging’ principles in the real world. Clinicians and service managers could find the changes in this model, which are reasonably simple and easy to implement, useful if they are trying to engage particular groups, such as specific racial and ethnic minorities [16].

We believe this model has the potential to reduce the prevalence of mental health problems by increasing the number of people in the community who are able to access evidence-based treatments, and with that, reduce the chronicity of problems. Additionally, it could have financial benefits [54?] as well as improved recruitment to research studies [3].

Several help-seeking models exist. We have mentioned the process model by Gask [8], which covers engagement/promotion and intervention aspects. Other well-known models of help-seeking, such as the ‘Behavioural Model of Health Services use’ [54], and the Theory of Planned Behaviour, focus on the attitudinal aspects prior to intervention [55].

## 4. Clinical Implications

Clinical services may fear being overwhelmed by demand if services are better publicised. However, this model complements existing services. A stepped care model already occurs with IAPT [56,57], or a staging process for early intervention is being developed, as relatively few people would need intensive treatment [58]. Those with no or mild symptoms could be signposted to self-help material or relevant websites, and those with more severe problems can be directed to more intensive services.

### Research Implications

Identifying how **‘PLACES’** factors could be applied to different clinical or demographic groups—and identifying the more relevant facilitators for these groups—would be valuable. For instance, normalisation has been found to be particularly important for boys/students in describing mental health problems, as they have fear of being seen as ‘weak’ [59]. Other groups where the **‘PLACES’** model could be applied may be south Asians [16] and Caribbean men [60]. For each of these groups, different methods for marketing and/or tailoring interventions could be compiled and systematically tested to see if the different methods result in better engagement. Treatment engagement scales can also be developed [61].

In order to make the best use of interventions that are clinically effective for people who need them, we should reduce the “treatment engagement gap” between treatments and seeking help. If the treatment engagement factors outlined in the **‘PLACES’** model are shown to be helpful with different groups, then these methods could be very helpful in reducing the prevalence of mental health problems and with it related benefits.

## Data Availability

Data availability is not applicable to this article as no new data were created or analysed in this study.
